# Pilot Study on Evaluating the Impact of Tetanus, Diphtheria, and Pertussis (Tdap), Influenza, and COVID-19 Vaccinations on Antibody Responses in Pregnant Women

**DOI:** 10.3390/vaccines12030312

**Published:** 2024-03-15

**Authors:** Wei-Chun Chen, Shu-Yu Hu, Chao-Min Cheng, Ching-Fen Shen, Hui-Yu Chuang, Chin-Ru Ker, Der-Ji Sun, Ching-Ju Shen

**Affiliations:** 1 Institute of Biomedical Engineering, National Tsing Hua University, Hsinchu 300, Taiwan; lionsmanic@gmail.com (W.-C.C.); chloe.natshuun@gmail.com (S.-Y.H.); chaomin@mx.nthu.edu.tw (C.-M.C.); 2 Division of Gynecologic Oncology, Department of Obstetrics and Gynecology, Chang Gung Memorial Hospital at Linkou, College of Medicine, Chang Gung University, Taoyuan 333, Taiwan; 3 Department of Obstetrics and Gynecology, New Taipei City Municipal Tucheng Hospital, New Taipei City 236, Taiwan; 4 International Intercollegiate Ph.D. Program, National Tsing Hua University, Hsinchu 300, Taiwan; 5 School of Traditional Chinese Medicine, Chang Gung University, Taoyuan 333, Taiwan; 6 Department of Pediatrics, National Cheng Kung University Hospital, College of Medicine, National Cheng Kung University, Tainan 701, Taiwan; drshen1112@gmail.com; 7 Department of Obstetrics and Gynecology, Kaohsiung Medical University Hospital, Kaohsiung Medical University, Kaohsiung 807, Taiwan; kawaiifish0517@yahoo.com.tw (H.-Y.C.); ruruk19@hotmail.com (C.-R.K.); 8 Graduate Institute of Clinical Medicine, College of Medicine, Kaohsiung Medical University, Kaohsiung 807, Taiwan; 9 Department of Obstetrics and Gynecology, Pojen Hospital, Kaohsiung 804, Taiwan; gummysun@gmail.com

**Keywords:** COVID-19 vaccine, neutralizing antibody, Nab, Tdap, pertussis, influenza, pregnancy, maternal vaccination

## Abstract

This study assessed IgG levels to influenza/pertussis and neutralizing antibody (Nab) responses of COVID-19 vaccines in blood of pregnant women following immunization with pertussis (Tdap), influenza, and COVID-19 vaccines. We prospectively collected 71 participants categorized by the following vaccine combinations: 3TI, 4TI, 3T, and 4T groups (three and four doses of COVID-19 vaccines plus Tdap/influenza or Tdap vaccines alone). Our findings have indicated that the 3TI group exhibited elevated IgG levels for influenza B compared to the 3T group (12.90 vs. 7.75 U, *p* = 0.001); this pattern was not observed for influenza A. Pertussis IgG levels remained uniform across all groups. The 4TI group demonstrated a greater Nab inhibition rate from COVID-19 vaccines compared to both the 3TI and 3T groups (61.34% vs. 22.5% and 15.16%, respectively, *p* = 0.001). We observed no correlation between Nab inhibition rate and IgG levels for Tdap/influenza, with the exception of a moderate correlation with influenza B in the 3TI group. The efficacy of Tdap vaccine in pregnant women remained consistent, regardless of the administration of COVID-19 or influenza vaccines. Interestingly, without the influenza vaccine, both three and four doses of the COVID-19 vaccine still offered protection against influenza A, but not B. Hence, co-administering COVID-19, influenza, and Tdap vaccines during prenatal care maintains immunogenicity and is highly advised to safeguard pregnant women fully.

## 1. Introduction

The COVID-19 pandemic, emerging in 2019, was caused by the SARS-CoV-2 virus. This pandemic, characterized by rapid viral spread, significantly strained healthcare systems and the worldwide economy [1]. It underscored the essential role that vaccinations play in controlling a virus’s dissemination and lessening the intensity of its effects, particularly for at-risk populations such as pregnant women [2]. Research indicates that pregnant women are at increased risk of severe complications such as preeclampsia and preterm births [3]. Maternal vaccination for COVID-19 not only protects these women but also, through the transfer of neutralizing antibodies across the placenta, offers protection to the fetus against the virus and its variants [4,5,6]. This approach emphasizes the critical role of vaccinations in protecting vulnerable populations, marking a substantial advancement in public health and scientific development amid the pandemic.

In our previously published research, it was observed that COVID-19 vaccination in pregnant women generated neutralizing antibodies that were transmitted to the fetus via transplacental transmission, and these transferred antibodies could function within the fetal system to provide protection against SARS-CoV-2 including various subvariants [4,5,6]. This evidence supports the administration of COVID-19 vaccines among pregnant women as a strategic measure to protect both mother and fetus. Following the American College of Obstetricians and Gynecologists (ACOG)’s guidelines, it is recommended that pregnant women receive both the seasonal influenza vaccine and the tetanus toxoid, reduced diphtheria toxoid, and acellular pertussis (Tdap) vaccines during the prenatal period [7,8]. The previously published literature demonstrated that antibodies produced in mothers as a result of these vaccinations can be also transmitted to their fetuses through transplacental transmission [9,10,11], as is the case with maternal COVID-19 vaccination. This transfer can provide the fetus with a level of immunity, highlighting the importance of maternal vaccination not just for the health of the mother, but also for offering an early protective barrier to the unborn child against certain infectious diseases.

ACOG has recommended the concurrent administration of COVID-19 and other vaccines, such as the influenza and Tdap vaccines, during pregnancy. However, the majority of COVID-19 vaccine trials have excluded participants who received other vaccinations concurrently with COVID-19 vaccines, leading to a paucity of data on potential vaccine interactions and the effects of co-vaccination. Due to the limited research exploring the potential impacts of administering these vaccines simultaneously, this study aims to investigate the effects of antibodies produced against types A and B influenza by the influenza vaccine and antibodies produced by the Tdap vaccine under various COVID-19 vaccine dosage scenarios. Furthermore, this research seeks to understand whether antibody production differs as a result of co-administration of influenza and Tdap vaccines under different vaccination scenarios.

## 2. Materials and Methods

### 2.1. Study Design and Participants Recruitment

This study was conducted at Kaohsiung Medical University Hospital and was carried out after receiving approval from the local institutional review board (IRB), with the designated IRB number: KMUHIRB-SV(II)-20210087. Our collected data encompassed pregnant women who had received at least three or four doses of COVID-19 vaccine, with the stipulation that the final dose must have been administered during the current pregnancy. Vaccines administered before pregnancy could include the Oxford/AstraZeneca ChAdOx1 nCoV-19 (AZD1222) vaccine, the Pfizer BioNTech (BNT162b2) COVID-19 vaccine, and the Spikevax (elasomeran) COVID-19 vaccine (previously called the mRNA-1273 Moderna vaccine). However, the COVID-19 vaccines administered during pregnancy were limited to the latter two mRNA-based COVID-19 vaccines. For those receiving a fourth dose of COVID-19 vaccine, the Moderna COVID-19 bivalent (SPIKEVAX Bivalent Original/Omicron BA.1 or BA.4/5) vaccine was used.

In addition to receiving a COVID-19 vaccine, all participating pregnant women were administered the Tdap vaccines (Adacel, Sanofi Pasteur, Toronto, ON, Canada) during their pregnancy. Moreover, a subset of these women also received the seasonal influenza vaccine (AdimFlu-S, QIS, Adimmune Corporation, Taichung, Taiwan; FlucelvaxQuad, CSL Behring GmbH, Marburg, Germany; VAXIGRIP TETRA, Sanofi Pasteur, Val-de-Reuil, Cedex, France). Based on the administration of three or four doses of COVID-19 vaccine, along with the Tdap and influenza vaccine or solely the Tdap vaccine, participants were categorized into four distinct groups. These included: (1) the group receiving three doses of COVID-19 vaccine and both Tdap and influenza vaccines (3TI); (2) the group receiving three doses of COVID-19 vaccine and only the Tdap vaccine (3T); (3) the group receiving four doses of COVID-19 vaccine and both Tdap and influenza vaccines (4TI); and, (4) the group receiving four doses of the COVID-19 vaccine and only the Tdap vaccine (4T). Comparisons were made across these groups.

All participants included in the study were required to meet the following criteria: singleton pregnancies; aged 20 or above; absence of preterm labor, symptoms associated with COVID-19/influenza or with previous COVID-19/influenza diagnosis history; no prior medical history indicating the need for immunosuppressant treatments. No significant discomfort or morbidity was reported after vaccination during the investigation.

### 2.2. Samples Collection

In this study, we enrolled pregnant women who met the specified eligibility criteria and provided informed consent before their delivery day during hospitalization. Maternal blood samples were collected from maternal peripheral blood during childbirth. All of the above obtained samples were sent to our laboratory for further analysis. We also gathered comprehensive clinical data from electronic medical records, including maternal age, parity, body mass index (BMI), neonatal weight and gender, vaccination dates for COVID-19, Tdap, and influenza, and gestational weeks at delivery. All of the above data were collected for further analysis.

### 2.3. Detection of IgG Antibody for Influenza Virus A, Influenza Virus B, and Pertussis by Enzyme-Linked Immunosorbent Assays (ELISA)

In this research, we evaluated the presence of IgG antibodies against influenza virus A, influenza virus B, and pertussis in maternal blood samples. Initially, the samples, which were stored frozen, were gently thawed at ambient temperature. Subsequently, we measured the levels of these antibodies using enzyme-linked immunosorbent assay (ELISA) techniques, adhering to the supplied protocols by the manufacturer (for influenza A: IgG Human ELISA Kit, No. ab108745, Abcam, Cambridge, United Kingdom; for influenza B: IgG Human ELISA Kit, No. ab108746, Abcam, Cambridge, United Kingdom; and for Bordetella pertussis: IgG Human ELISA Kit, No. ab108709, Abcam, Cambridge, United Kingdom). Samples were diluted at a ratio of 1:100 using the provided IgG sample diluent. Following the preparation of all necessary reagents and controls according to the manufacturer’s instructions, samples and controls were allocated to the assay wells and plates were incubated at 37 °C for one hour. This step was followed by a washing process, after which a prepared horseradish peroxidase (HRP)-conjugated label was added and left to sit at room temperature for 30 min. Following another wash, TMB (3,3′,5,5′-tetramethylbenzidine) substrate solution was introduced into each well and left to react at room temperature for 15 min. The reaction was halted by adding a stop solution. The optical density of each well was then determined at a wavelength of 450 nm within 30 min after the stop solution was applied, utilizing a standard curve to convert these readings into specific antibody concentrations for the analyzed samples.

### 2.4. The Neutralizing Antibody Inhibition Test of SARS-CoV-2 Omicron BA.5

Using the ELISA assay with the kit from Acro Biosystems (Cat. No. RAS-N107, Newark, DE, USA), we measured the inhibition rate of neutralizing antibodies against the SARS-CoV-2 Spike RBD in our samples. The neutralizing antibody inhibition rate was detected and evaluated through a competitive ELISA utilizing a solution containing the spike protein receptor-binding domain (SRBD). This SRBD solution was designed to engage with the neutralizing antibody present in our samples and the angiotensin-converting enzyme 2 (ACE2) protein, which had been previously applied to the surface of 96-well microplates. Initially, the microplates were loaded with both the samples and controls, followed by the introduction of a horseradish peroxidase (HRP)-conjugated SRBD solution, catering to different SARS-CoV-2 variants. The assay entailed a one-hour incubation at ambient temperature, protected from light exposure. Subsequent to this incubation, the supernatant was discarded, and the plates were washed to remove unbound substances. A substrate solution was then added to each well, followed by another 20 min of incubation in darkness. The reaction was halted by the addition of a stop solution, causing a color change in the wells, which signified the reaction’s completion. Absorbance values at 450 nm/630 nm were subsequently determined using an ELISA reader.

### 2.5. Statistics

A chi-square test was utilized to assess the differences in antibody proportions among the study participants receiving different doses of COVID-19 vaccines or different prenatal vaccination including Tdap or influenza vaccines. These participants were subsequently organized into various subgroups based on these proportions. To examine the disparities in IgG antibody levels against influenza A, influenza B, or pertussis across these subgroups, we applied analysis of variance (ANOVA) and sample *t*-tests. Additionally, we investigated the association between IgG antibody levels against influenza A, influenza B, or pertussis and their relations with neutralizing antibody inhibition rate by employing the Pearson correlation coefficient. Data analysis and processing were carried out using SPSS Statistics (version 27, IBM, Armonk, NY, USA) and Microsoft Excel (version 16.8, Microsoft, Redmond, WA, USA), with a *p*-value of less than 0.05 deemed to indicate statistical significance. The visualization of these statistical outcomes was facilitated via GraphPad Prism software (version 9, GraphPad Software, San Diego, CA, USA).

## 3. Results

### 3.1. Participants Characteristics

In this study, blood samples from a total of 71 participants were collected, including 53 who had received three doses of the COVID-19 vaccine and 18 who had received four doses. The characteristics of the participants are presented in Table 1. The average age was around 34 years, with approximately 18–22% of the participants being primiparous, the remainder being multiparous, and about 5–7% having had three parities prior to the current gestation. The mean gestational age at delivery was between 38 and 39 weeks, with a mean BMI of approximately 25–27. The mean neonatal birth weight was between 3050 and 3100 g, with male newborns accounting for about 50–55% and females for 45–50%. Between the cohorts who received three or four doses of COVID-19 vaccine, the characteristics were mostly similar, except for the significant differences in the proportions of those vaccinated with Tdap and the influenza vaccine, and those vaccinated with Tdap only. The proportion of participants vaccinated with both Tdap and the influenza vaccine was 39.6% among those who received three doses of the COVID-19 vaccine, but it was significantly higher at 83.3% among those who received four doses (*p* = 0.001). This discrepancy may be attributed to the smaller number of cases in the four-dose cohort, which could lead to larger variations. Other than this, the basic composition of the groups showed no difference.

Table 2 presents the levels of anti-influenza A and anti-influenza B IgG, as well as anti-pertussis IgG, across various scenarios including the four groups previously mentioned: 3TI, 3T, 4TI, and 4T. The following sections will present the comparisons in antibody levels among these groups.

### 3.2. IgG of Influenza A, B and Pertussis Levels between 3 and 4 Doses of COVID-19 Vaccination in Our Cohort

**Figure 1:**
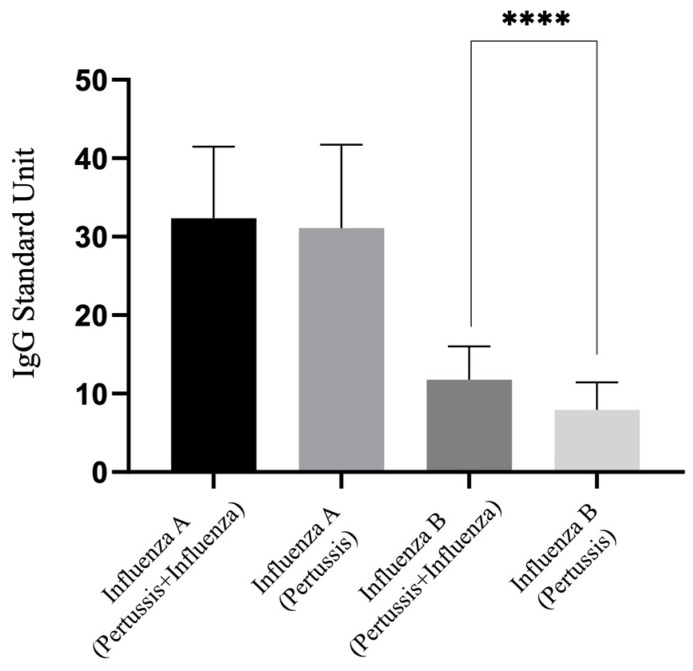
IgG levels for influenza A and B among participants vaccinated with three or four doses of the COVID-19 vaccine: impact of pertussis/influenza versus pertussis-only vaccination. ****, *p* < 0.0001.

Figure 1 illustrates the differences in IgG antibodies for influenza A and influenza B among all participants, comparing those vaccinated with both Tdap and the influenza vaccine to those vaccinated with only the Tdap vaccine, essentially comparing 3TI/4TI versus 3T/4T groups. It was observed that IgG levels for influenza B were significantly lower in the absence of the influenza vaccination, indicating a statistically significant difference. However, no difference was found in IgG levels for influenza A between the two groups.

As also indicated in Table 2, there was a difference in IgG levels for influenza B between the 3TI and 3T groups (12.90 vs. 7.75 U, *p* = 0.001). Interestingly, no significant difference was found in IgG levels for influenza B between the 4T group and either the 3TI or 4TI groups (4T vs. 3TI: *p* = 0.639; 4T vs. 4TI: *p* = 1.000). Similarly, no difference was observed in IgG levels for influenza A when comparing the 3T or 4T groups with the 3TI or 4TI groups (3T vs. 3TI: *p* = 0.926; 3T vs. 4TI: *p* = 0.995; 4T vs. 3TI: *p* = 0.493; 4T vs. 4TI: *p* = 0.757).

For Nab inhibition rate, there was no significant difference between the 4T group and both the 3TI and 3T groups (*p* = 0.449 and *p* = 0.163, respectively). Similarly, no significant difference was observed when comparing the 4T group with the 4TI group (*p* = 0.464), which could be attributed to the smaller sample size of the 4T group. In contrast, the group with a larger sample size, 4TI, showed a significantly higher Nab inhibition rate compared to both the 3TI and 3T groups (both *p* = 0.001).

### 3.3. IgG of Influenza A, B and Pertussis Levels between 3 and 4 Doses of COVID-19 Vaccination in Those Receiving Tdap Plus Influenza Vaccines

**Figure 2:**
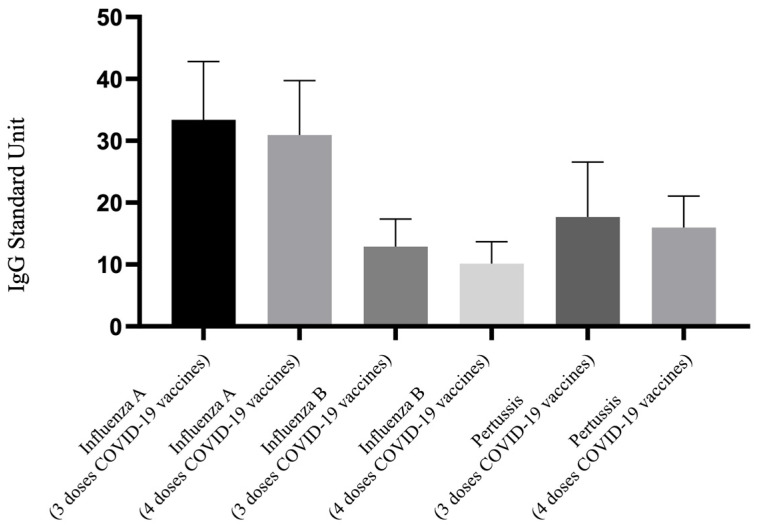
Comparison of IgG antibody to influenza A/B and pertussis for participants that received 3 doses and 4 doses of COVID-19 vaccine and Tdap and influenza vaccine. Tdap, the tetanus toxoid, reduced diphtheria toxoid, and acellular pertussis.

Figure 2 demonstrates the differences in IgG antibody levels against influenza A, influenza B, and pertussis among pregnant women who received both the Tdap and influenza vaccines, under the regimen of three versus four doses of the COVID-19 vaccine. This comparison between the 3TI and 4TI groups focuses on the antibody levels for these three target vaccines. The visual representation indicates no discernible difference in the levels of these three IgG antibodies between the 3TI and 4TI groups. According to the data presented in Table 2, there is no significant difference in the comparison between the 3TI and 4TI groups regarding their antibody levels (influenza A IgG: 33.4 vs. 30.93 U, *p* = 0.881; influenza B IgG: 12.90 vs. 10.14 U, *p* = 0.148; pertussis IgG: 17.69 vs. 15.98 U, *p* = 0.911). However, for Nab inhibition rate, the 4TI group exhibited a higher Nab inhibition rate than the 3TI group (61.34% vs. 22.50%, *p* = 0.001), indicating a significant increase in Nab inhibition rate with the administration of an additional dose of the COVID-19 vaccine.

### 3.4. IgG of Influenza A, B and Pertussis Levels between 3 and 4 Doses of COVID-19 Vaccination in Those Receiving Further Tdap Vaccine Alone

**Figure 3:**
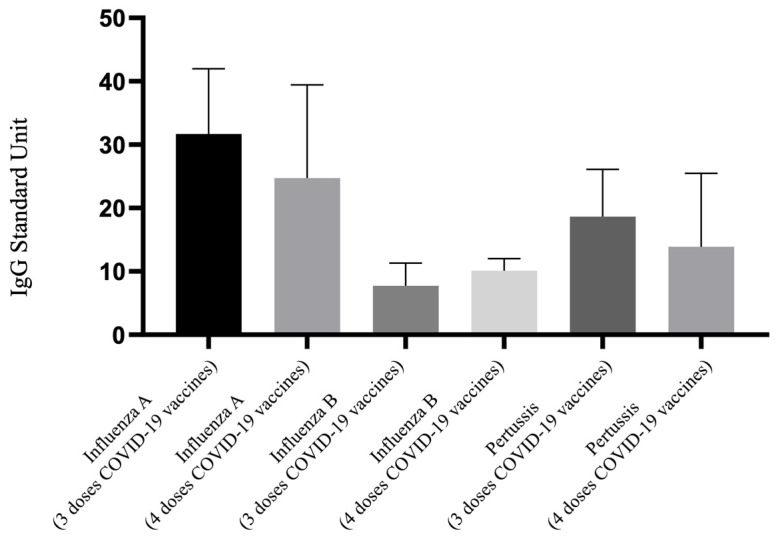
Comparison of IgG antibody to influenza A/B and pertussis for participants that received 3 doses and 4 doses of COVID-19 vaccine and Tdap vaccine alone. Tdap, the tetanus toxoid, reduced diphtheria toxoid, and acellular pertussis.

Figure 3 illustrates the differences in IgG antibody levels against influenza A, influenza B, and pertussis among pregnant women who were vaccinated solely with the Tdap vaccine, comparing those who received three versus four doses of the COVID-19 vaccines. This analysis focuses on the differences between the 3T and 4T groups. The graphical representation shows that there was no significant difference in the antibody levels for these three pathogens between the two groups. Data from Table 2 corroborate this observation, showing no significant disparities in antibody levels between the 3T and 4T groups (influenza A IgG: 31.68 vs. 24.74 U, *p* = 0.653; influenza B IgG: 7.75 vs. 10.12 U, *p* = 0.731; pertussis IgG: 18.64 vs. 13.89 U, *p* = 0.734). Regarding Nab inhibition rate, the 4T group displayed a higher percentage compared to the 3T group (41.85% vs. 15.16%, *p* = 0.163); although, this did not reach statistical significance, possibly due to the sample size of the 4T group.

### 3.5. IgG of Pertussis Levels between Tdap with or without Further Influenza Vaccines in Those Receiving 3 and 4 Doses of COVID-19 Vaccination

**Figure 4:**
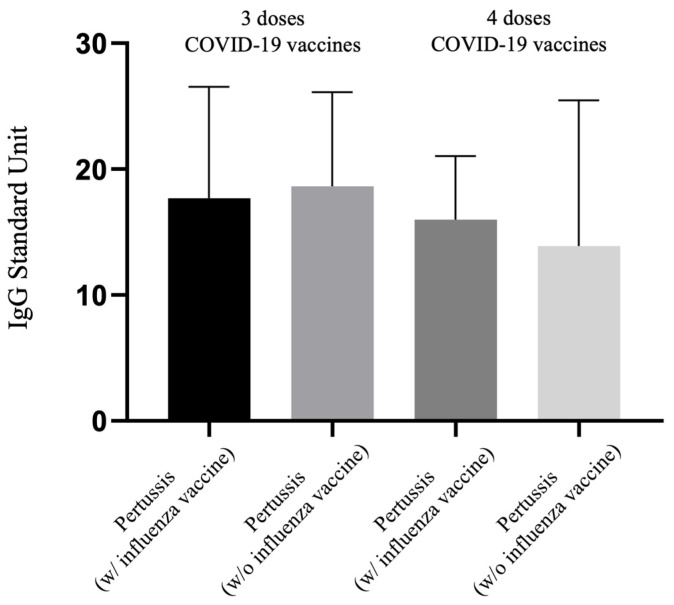
Comparison of IgG antibody to pertussis for participants receiving 3 or 4 doses of COVID-19 vaccine with or without antenatal influenza vaccination.

Figure 4 compares the levels of pertussis IgG antibodies among pregnant women vaccinated with the same regimen of three or four doses of COVID-19 vaccine, differentiating between those who received both the Tdap and influenza vaccines and those who were vaccinated with only the Tdap vaccine. This entails a comparison between the 3TI vs. 3T and 4TI vs. 4T groups in terms of pertussis IgG levels. The graphical analysis indicates no significant differences in the pertussis IgG values across these comparisons. Data presented in Table 2 also support this finding, showing no statistically significant difference in pertussis IgG levels between the 3TI and 3T groups (17.69 vs. 18.64 U, *p* = 0.971) or between the 4TI and 4T groups (15.98 vs. 13.89 U, *p* = 0.973). Similarly, no significant differences were observed in neutralizing antibody (Nab) inhibition rate among these comparisons (3TI vs. 3T: 22.50% vs. 15.16%, *p* = 0.601; 4TI vs. 4T: 61.34% vs. 41.85%, *p* = 0.464), indicating that the additional immunization against influenza does not significantly alter the pertussis IgG levels or Nab inhibition rate among these groups of pregnant women.

### 3.6. Correlation among Nab Inhibition Rates to SARS-CoV-2 and IgG Antibody to Influenza A/B and Pertussis in Those with 3 or 4 Doses of COVID-19 Vaccines with Further Vaccination of Tdap Plus Influenza or Tdap Alone

**Figure 5:**
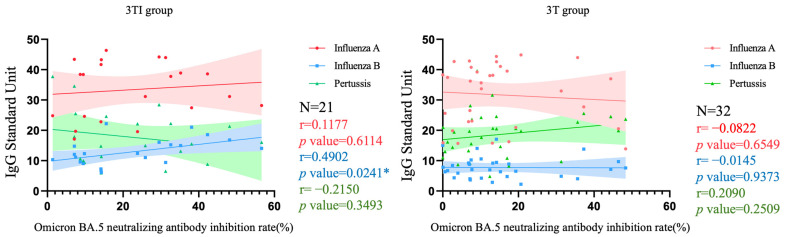
Correlation among Nab inhibition rates to Omicron BA.5 SARS-CoV-2 and IgG antibody to influenza A/B and pertussis in participants that received 3 doses of COVID-19 vaccine and Tdap/influenza or Tdap alone vaccination. 3TI group, 3 doses of COVID-19 vaccines plus Tdap/influenza vaccines; 3T group, 3 doses of COVID-19 vaccines plus Tdap vaccine; Nab, neutralizing antibody; Tdap, the tetanus toxoid, reduced diphtheria toxoid, and acellular pertussis; *, *p* < 0.05.

**Figure 6:**
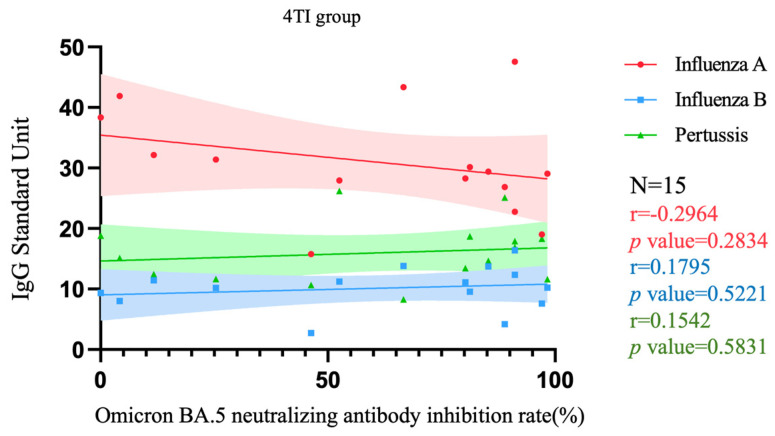
Correlation among Nab inhibition rates to Omicron BA.5 SARS-CoV-2 and IgG antibody to influenza A/B and pertussis in participants that received 4 doses of COVID-19 vaccine and Tdap/influenza vaccination. 4TI group, 4 doses of COVID-19 vaccines plus Tdap/influenza vaccines; Nab, neutralizing antibody; Tdap, the tetanus toxoid, reduced diphtheria toxoid, and acellular pertussis.

Figure 5 and Figure 6, respectively, depict the correlation between Nab inhibition rate and levels of three target antibodies among pregnant women who received three doses of the COVID-19 vaccine and were either vaccinated with both Tdap and influenza vaccines (denoted as 3TI) or only with the Tdap vaccine (denoted as 3T), as well as among pregnant women who received four doses of the COVID-19 vaccine and were vaccinated with both Tdap and influenza vaccines (denoted as 4TI). In the 3TI group, a significant correlation was only observed between influenza B IgG levels and Nab inhibition rate (*p* = 0.0241), with an r value of 0.4902 indicating a moderate positive correlation between these two variables. The correlations of other antibodies were not significant, with r values indicating low correlation (all < 0.40). In the 3T group, none of the antibodies showed significant correlations, and all r values were indicative of low correlation (all <0.40). Similarly, in the 4TI group, there were no significant correlations observed for all antibodies, with r values also suggesting low correlation (all < 0.40).

## 4. Discussion

From our research, several things are evident. Although the 3TI group, compared to the 3T group, received the influenza vaccine and thus exhibited higher levels of influenza B IgG, no such increase was observed in influenza A IgG levels in the 3TI group relative to the 3T group, nor were increased levels observed in the 4TI group compared to either the 3T or 4T groups. This suggests that receiving at least three doses of the COVID-19 vaccine, even without the influenza vaccine, might offer indirect protection against influenza in pregnant women. For pertussis IgG levels, no differences were observed across any comparisons, indicating that vaccination with the Tdap vaccine, regardless of the number of COVID-19 vaccine doses received or whether the influenza vaccine was administered, does not affect the protective effect against pertussis in pregnant women.

Regarding Nab inhibition rate to SARS-CoV-2 achieved via receipt of COVID-19 vaccine, the 4TI group, which received four doses of the COVID-19 vaccine, exhibited higher Nab inhibition rate than groups that only received three doses (3TI or 3T). This is consistent with previous study findings. However, there was no significant difference between the 4T group and the 3TI or 3T groups, nor between the 4T and 4TI groups, which could be attributed to the smaller sample size of the 4T group. Regarding the correlation between Nab inhibition rate to SARS-CoV-2 from the COVID-19 vaccine and the levels of the three target antibodies, there was generally no correlation between the COVID-19 vaccine’s Nab inhibition rate and the three IgG levels for Tdap and influenza vaccines. However, a moderate positive correlation was found between the protective effect against influenza B and the Nab inhibition rate from the COVID-19 vaccine. As previously described, this suggests that receiving at least three doses of the COVID-19 vaccine might also offer indirect protection against influenza in pregnant women.

Based on the published literature, patients co-infected with SARS-CoV-2 and influenza virus are approximately twice as likely to die compared to those infected solely with SARS-CoV-2 [12]. It has been suggested that the influenza virus increases the expression of angiotensin-converting enzyme 2 (ACE2), facilitating the entry of SARS-CoV-2 into cells [13]. Previous studies also indicate a negative correlation between influenza vaccination rates and COVID-19 mortality [14], and it has been noted that those vaccinated against influenza show a decrease in COVID-19 infection rates [15]. The influenza virus contains CD8+ cell peptides that can cross-react with SARS-CoV-2’s ACE2, thus antibodies produced in response to influenza virus activation can offer cross-protection against SARS-CoV-2 [16]. Additionally, the response to H1N1 CD4+ is strongly positively correlated with SARS-CoV-2-specific CD4+ cells, suggesting an associative response between the two [17]. Therefore, cross-reactive immune responses present in individuals prior to SARS-CoV-2 exposure may also affect susceptibility to infection and disease severity. This situation, apart from influenza virus, can also be seen in Japanese encephalitis virus infections where cross-reactive T cell responses can offer protection against SARS-CoV-2 [18]. This indirectly shows that influenza vaccines might mitigate the impact of COVID-19 through vaccine-induced changes in innate immunity and cross-reactivity responses [19,20,21].

Conversely, studies have indicated that vaccination against COVID-19 can also increase antibody levels against influenza proteins, including IgG to spike protein and neuraminidase protein [22]. Therefore, the increase in immunity to influenza infection following COVID-19 vaccination, although its mechanism is not clear, may partly be explained by this vaccine’s cross-protection which reduced incidence of influenza infection during this pandemic; however, this reduction in incidence also may reflect the effects of public health quarantine measures [23]. Our study also revealed that pregnant women who received a sufficient number of COVID-19 vaccine doses, even without the administration of the influenza vaccine, still exhibited protection against influenza A. However, protection against influenza B may not be as robust.

Pertussis, also known as whooping cough, is an acute respiratory infection caused by Bordetella pertussis, affecting individuals across all age groups, though predominantly impacting children. Notably, severe disease manifestations are particularly concerning in infants under three months of age [24,25]. The administration of the Tdap (tetanus, diphtheria, acellular pertussis) vaccine to pregnant women is a critical strategy for safeguarding newborns from the severe repercussions of pertussis during the initial months of life [26,27]. Observations during the COVID-19 pandemic have highlighted a significant reduction in pertussis case numbers, correlating with the stringent measures implemented to curb the spread of COVID-19 [28]. This sustained reduction in pertussis cases, primarily seen in infants and children, is likely attributed to the preventive measures against COVID-19 transmission. School and daycare closures, along with social distancing and mask-wearing mandates, have potentially led to a marked decrease in pertussis cases among children and infants, subsequently reducing transmission to adults responsible for their care [29,30].

To date, there has been no literature documenting any interference of the Tdap vaccine with the immunogenicity or protective efficacy of COVID-19 or influenza vaccines. Our study indicates that, among pregnant women who have received either three or four doses of vaccines, no significant difference was observed in pertussis IgG levels between those vaccinated with Tdap/influenza and influenza alone. Similarly, a low correlation was found with the Nab inhibition rate of SARS-CoV-2, suggesting that pregnant women receiving the Tdap vaccine need not be concerned about interference with the efficacy of COVID-19 or influenza vaccines.

Maternal immunization serves as a critical strategy to offer protection against the sequelae of a variety of infectious diseases for newborns, mothers, or both [8,31]. Currently, the World Health Organization (WHO) recommends that pregnant women receive vaccinations against pertussis, COVID-19, and influenza to safeguard newborns from severe pertussis in their initial months of life, as well as to protect both pregnant women and newborns from severe outcomes of influenza and COVID-19 [7,8]. However, the potential interference between these vaccines in terms of immunogenicity or protective efficacy in pregnant women has been scarcely discussed. Our study indicates that the prevention of pertussis is not influenced by whether pregnant women receive the influenza vaccine or various doses of the COVID-19 vaccine. Pertussis and SARS-CoV-2 are transmitted via respiratory droplets, with pertussis being far more contagious than SARS-CoV-2 [32,33]. Thus, the continued promotion of Tdap vaccination among pregnant women, without significant interference among vaccines, is necessary.

For protection against influenza, pregnant women who received three or four doses of the COVID-19 vaccine, even without receiving the influenza vaccine, still maintained protection against influenza A comparable to those vaccinated against influenza. However, pregnant women without the influenza vaccine showed insufficient protection against influenza B. Therefore, vaccination against influenza during pregnancy is essential to maintain adequate protection against the influenza virus. Regarding the neutralizing antibody inhibition rate of SARS-CoV-2 induced by the COVID-19 vaccine, we observed that a higher number of vaccine doses corresponded with a greater neutralizing antibody inhibition rate. Furthermore, there was no correlation between pertussis antibodies, influenza A antibodies, and neutralizing antibody inhibition rates. Figure 5 and Figure 6 depict the analysis, focusing on groups with a higher number of participants who received three doses of the COVID-19 vaccine, specifically the 3T and 3TI groups, while excluding the smaller 4T and 4TI groups. The relation between influenza B and the neutralizing antibody inhibition rate shows a significant *p*-value, indicating a moderate positive correlation. This suggests that individuals vaccinated against influenza, with higher IgG levels for influenza B, are likely to exhibit a higher neutralizing antibody inhibition rate. This correlation implies no negative interactions between the vaccines. Additionally, previous research also suggested that the combined administration of the COVID-19 vaccine and the influenza vaccine is safe [34,35]. Therefore, as per the WHO recommendation, combined vaccination was a feasible option to increase coverage rates [36].

Our study faced several limitations. Firstly, there was an uneven distribution among participants regarding the doses and combinations of vaccines administered. Specifically, 53 participants received three doses of the COVID-19 vaccine, whereas only 18 received four doses. This imbalance extended to the composition of participants vaccinated with both Tdap/influenza and those receiving only the Tdap vaccine. For instance, among those who got four doses of the COVID-19 vaccine, only three cases (16.7%) were exclusively given the Tdap vaccine, compared to 60% in the three-dose group. Such disparities in distribution and sample size could influence the study outcomes, highlighting an area for improvement in future recruitment efforts. Moreover, participants may have received different COVID-19 vaccines before and during their pregnancy, such as the Oxford/AstraZeneca ChAdOx1 nCoV-19 (AZD1222) vaccine followed by either the Pfizer BioNTech (BNT162b2) or Spikevax (elasomeran), previously known as the Moderna mRNA-1273 vaccine. Given that all these vaccines are WHO-approved, the variation in vaccination regimens could introduce bias into our findings. Additionally, the high rate of antenatal Tdap vaccine administration in Taiwan resulted in the absence of Tdap-unvaccinated pregnant women in our study, further limiting our research and suggesting an avenue for recruitment enhancement in future studies.

## 5. Conclusions

In our study, we have demonstrated that protective efficacy against pertussis following Tdap vaccination was not influenced by whether the recipient had been administered three or four doses of the COVID-19 vaccine or whether they had received the influenza vaccine. Pregnant women who had received three or four doses of the COVID-19 vaccine, without the influenza vaccine, may still maintain protection against influenza A; however, their protection against influenza B was found to be insufficient. The neutralizing antibody (Nab) inhibition rate of SARS-CoV-2 showed no correlation with antibodies against pertussis and influenza A, but it exhibited a moderate positive correlation with antibodies against influenza B. Therefore, it is still recommended that pregnant women receive COVID-19, influenza, and Tdap vaccines during antenatal care. This approach ensures comprehensive and full protection for both pregnant women and newborns without any negative immunogenic interference among the vaccines.

## Figures and Tables

**Table 1:** Participant characteristics.

	3 Doses COVID-19 Vaccines (*n* = 53)	4 Doses COVID-19 Vaccines (*n* = 18)	*p* Value
Mean age (range) (years)	34.13 (22–45)	34.06 (27–43)	0.954
Parity, N (%)			0.981
0	10 (18.9)	4 (22.2)	
1	20 (37.7)	7 (38.9)	
2	19 (35.8)	6 (33.3)	
3	4 (7.5)	1 (5.6)	
Mean gestational weeks (range)	38.52 (36–40)	39.0 (38–40)	0.109
Mean BMI (range)	27.54 (20.45–38.58)	25.95 (21.64–33.82)	0.155
Mean neonatal BW (range) (gm)	3099.34 (2340–3855)	3068.89 (2325–3645)	0.730
Neonatal gender, N (%)			0.475
Male	27 (50.9)	10 (55.6)	
Female	26 (49.1)	8 (44.4)	
Antenatal vaccination, N (%)	2		0.001
Tdap + Influenza	1 (39.6)	15 (83.3)	
Tdap	32 (60.4)	3 (16.7)	

BMI, body mass index; BW, body weight; Tdap, the tetanus toxoid, reduced diphtheria toxoid, and acellular pertussis.

**Table 2:** Levels of IgG against influenza A, B, and pertussis levels, and Nab inhibition rates of COVID-19 vaccines.

	3TI Group (*n* = 21)	3T Group (*n* = 32)	4TI Group (*n* = 15)	4T Group (*n* = 3)	*p* Value
IgG to Influenza A (U)	33.40 (17.09–46.34)	31.68 (13.91–44.87)	30.93 (15.78–47.56)	24.74 (11.11–40.29)	0.537 (total) 0.926 (3TI vs. 3T) 0.881 (3TI vs. 4TI) 0.493 (3TI vs. 4T) 0.995 (3T vs. 4TI) 0.653 (3T vs. 4T) 0.757 (4TI vs. 4T)
IgG to Influenza B (U)	12.90 (6.09–22.19)	7.75 (2.24–17.07)	10.14 (2.74–16.39)	10.12 (7.91–11.26)	0.001 (total) 0.001 (3TI vs. 3T) 0.148 (3TI vs. 4TI) 0.639 (3TI vs. 4T) 0.196 (3T vs. 4TI) 0.731 (3T vs. 4T) 1.000 (4TI vs. 4T)
IgG to Pertusis (U)	17.69 (5.97–37.75)	18.64 (4.80–39.59)	15.98 (8.26–26.17)	13.89 (7.10–27.27)	0.582 (total) 0.971 (3TI vs. 3T) 0.911 (3TI vs. 4TI) 0.852 (3TI vs. 4T) 0.685 (3T vs. 4TI) 0.734 (3T vs. 4T) 0.973 (4TI vs. 4T)
Nab inhibition rate of COVID-19 vaccines (%)	22.50 (1.38–56.63)	15.16 (0–48.36)	61.34 (0–98.29)	41.85 (12.76–56.58)	0.001 (total) 0.601 (3TI vs. 3T) 0.001 (3TI vs. 4TI) 0.449 (3TI vs. 4T) 0.001 (3T vs. 4TI) 0.163 (3T vs. 4T) 0.464 (4TI vs. 4T)

3TI group, 3 doses of COVID-19 vaccines plus Tdap/influenza vaccines; 3T group, 3 doses of COVID-19 vaccines plus Tdap vaccine; 4TI group, 4 doses of COVID-19 vaccines plus Tdap/influenza vaccines; 4T group, 4 doses of COVID-19 vaccines plus Tdap vaccine; Nab, neutralizing antibody; Tdap, the tetanus toxoid, reduced diphtheria toxoid, and acellular pertussis.

## Data Availability

All data can be obtained in the published paper.
